# Ecological responses of phytoplankton and bacterial communities to eutrophication in the Han River Basin

**DOI:** 10.3389/fmicb.2025.1649806

**Published:** 2026-01-05

**Authors:** Yuanyuan Chen, Fangtao Cai, Zhiyuan Qi, Tianqi He, Jiao Fang, Dongdong Zhai, Hongyan Liu, Ming Xia, Zhangfeng Hu, Yanfu Que, Fei Xiong, Bin Zhu

**Affiliations:** 1 Hubei Engineering Research Center for Protection and Utilization of Special Biological Resources in the Hanjiang River Basin, School of Life Sciences, Jianghan University, Wuhan, China; 2 School of Life Sciences, Institute of Microalgae Synthetic Biology and Green Manufacturing, Jianghan University, Wuhan, China; 3 Hubei Key Laboratory of Environmental and Health Effects of Persistent Toxic Substances, Jianghan University, Wuhan, China; 4 Institute of Hydroecology, Ministry of Water Resources and Chinese Academy of Sciences, Wuhan, China

**Keywords:** eutrophication, aquatic ecosystems, phytoplankton-bacteria interaction, Han River, ecological networks, water quality management

## Abstract

**Introduction:**

The Han River Basin, a critical water source for the South-to-North Water Diversion Project, faces escalating eutrophication pressures due to intensive anthropogenic activities. This study aims to systematically evaluate the ecological responses of phytoplankton and bacterial communities to eutrophication gradients, and to elucidate their interactions for guiding ecosystem restoration.

**Methods:**

Water samples were collected from 15 sites across the Han River Basin in July 2023. Phytoplankton composition was identified microscopically (6 phyla, 33 genera), and bacterial communities were characterized via high-throughput sequencing of the 16S rRNA gene. Trophic states were assessed using a modified Carlson’s Trophic State Index (TSI). Relationships between environmental variables (TN, TP, Chl-a, COD, etc.) and community structures were analyzed via Monte Carlo tests, redundancy analysis (RDA), and co-occurrence network analysis.

**Results:**

Eutrophication Status: 75% of sites were eutrophic (TSI: 42.5–66.0), with significant spatial variations in TN (1.10–6.00 mg/L), TP (0.010–0.29 mg/L), and Chl-a (0.86–70.00 μg/L). Community Shifts: Phytoplankton dominance transitioned from Bacillariophyta in low-TSI areas to Cyanophyta in high-TSI regions. Bacterial communities were dominated by Proteobacteria (>60% abundance), with diversity declining as eutrophication intensified. Environmental Drivers: Monte Carlo tests indicated Chl-a and COD as key drivers for bacteria (*p* < 0.05), while TN primarily influenced phytoplankton (*r* = 0.39, *p* < 0.01). Network Interactions: Co-occurrence networks revealed increased negative correlations (0.32% to 0.61%) and reduced modularity (0.641 to 0.558) under eutrophic conditions, suggesting intensified competition.

**Conclusion:**

Eutrophication filters species adaptability, leading to deterministic succession in phytoplankton and homogenization of bacterial communities. The rise in negative correlations underscores escalating resource competition, potentially destabilizing ecosystem functions. Our findings emphasize the urgency of nutrient load reduction and adaptive management. Future studies should prioritize leveraging phytoplankton-bacterial synergism for bioremediation and resilience enhancement.

## 1 Introduction

In community ecology, a primary objective is to comprehend how community composition shifts across different spatial scales (Whittaker, 1972; Liu et al., 2024). Eutrophication stands out as a major force shaping community dynamics (Reynolds, 1984; Jin et al., 2023). It often diminishes environmental diversity and excludes species that are sensitive to eutrophic conditions, such as those affected by shading, hypoxia, elevated ammonia levels, and reduced zooplankton grazing (Reynolds, 1984). This process can result in biotic homogenization, particularly at the local level (Rahel, 2002; Cavalcante et al., 2023; Xiang et al., 2023), which may in turn degrade biodiversity and the functionality of freshwater ecosystems (Rahel, 2002; Xiang et al., 2023). To counteract these effects, numerous countries have implemented measures to decrease external nutrient inputs to foster ecosystem restoration (Coveney et al., 2005; Xu et al., 2025). However, the outcomes of reducing both external and internal nutrient loads can differ significantly based on the specific strategies employed and their influence on the local abiotic and biotic community composition (Lauridsen et al., 2003; Chen et al., 2020; Polauke et al., 2024).

In aquatic ecosystems, phytoplankton serve as essential primary producers (Falkowski, 1994), and their response to pollutants is magnified through the nutrient cycle. Bacteria, which make up nearly 25% of the photic zone’s total biomass in natural waters, play a crucial role in regulating ecosystem cycles (Fu et al., 2020). The symbiotic relationship between phytoplankton and bacteria, evident since their early evolution, forms a fundamental microecological interaction in aquatic environments (Li et al., 2023). Phytoplankton offer attachment sites for bacteria and supply oxygen and organic carbon through photosynthesis, while bacteria provide inorganic nutrients that support phytoplankton growth (Fouilland et al., 2014). This close physical and nutritional interplay makes the coexistence of phytoplankton and bacteria a common phenomenon. Research indicates that both phytoplankton and bacteria are highly sensitive to environmental changes (Geng et al., 2022). As integral components of the food web, their interactions with local environmental processes render them valuable indicators for assessing pollution. Additionally, phytoplankton and bacteria are ideal model organisms for studying ecological responses. While single-species testing systems are commonly used to evaluate environmental contamination risk and pollutant toxicity, they fail to reflect the complex interactions seen in natural ecosystems (Bellingeri et al., 2024). Therefore, to more accurately assess the impact of pollutants on aquatic ecosystems, multi-species systems have gained increasing attention (Carraturo et al., 2024).

Earlier research has delved into how phytoplankton and bacterial communities shift in polluted settings. Harmful algal blooms tend to bolster the cooperative link and mutualistic interplay between algal and bacterial groups (Xu et al., 2022). Compared to standalone phytoplankton or bacteria, the combined phytoplankton-bacterial communities offer a more precise means of gaging ecological risks and indicate more effective at eliminating pollutants for water purification purposes (Geng et al., 2022). These benefits are particularly notable when it comes to assessing and tackling eutrophication pollution. Yet, there remains a gap in our grasp of the algal-bacterial assemblage in areas affected by eutrophication. We hypothesize that (i) eutrophication drives a deterministic shift from diatom- to cyanobacteria-dominated phytoplankton; (ii) this shift restructures bacterial communities, increasing network connectivity and negative interactions; and (iii) these community-level responses can be predicted from combined nutrient and physical variables. The Han River, a major tributary of China’s Yangtze River, is a vital water supply for the Middle Route of the South-to-North Water Diversion Project. The Han River Basin has seen swift urban expansion, intense farming, and robust economic activity, all of which have reshaped the ecological landscape and likely affected water quality (Lv et al., 2023), with eutrophication problems growing more acute. For example, Xin et al. (2020) pointed out that harmful algal blooms in the middle and lower sections of the Han River have been a major concern for drinking water quality since the 1990s.

This research involved gathering water samples from 15 distinct locations within the Han River Basin and tracking the physical and chemical properties of the water. The makeup and quantities of phytoplankton across varying degrees of pollution were identified. Employing high-throughput sequencing techniques, we examined the composition and diversity of bacterial communities, shedding light on how spatial pollution differences shape phytoplankton and bacterial characteristics. We also mapped out the community structures of phytoplankton and bacteria and probed into how nitrogen and phosphorus fluctuations correlate with pollution at various scales. The study is designed to address three key questions: (1) Is there a gradient-like shift in eutrophication pollution levels throughout the Han River Basin? (2) How do phytoplankton and bacterial communities react to eutrophication? (3) What influence does eutrophication have on the relationship between phytoplankton and bacteria? Co-occurrence networks were used to reveal potential interaction hubs that could serve as early-warning bio-indicators for nutrient management in the Han River Basin. Ultimately, the findings will enhance our grasp of how phytoplankton and bacteria interact in eutrophication-affected settings and offer theoretical backing for refining eutrophication bioremediation strategies.

## 2 Experimental design and methods

### 2.1 Description of the study location

**Figure 1:**
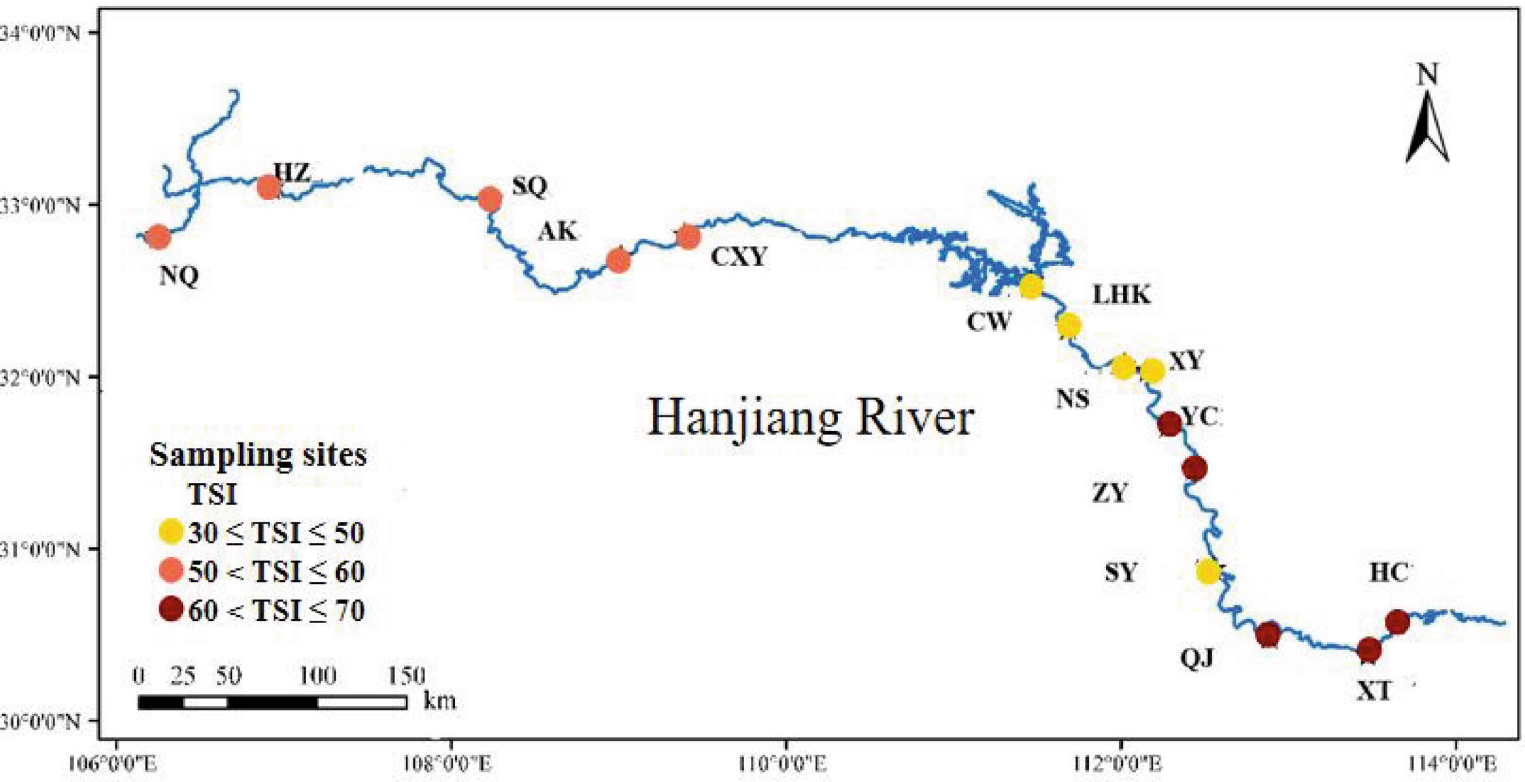
Sampling sites on the Hanjiang River, with each site labeled according to its section: H for high trophic (YC, ZY, QJ, XT, HC), M for medium trophic (NQ, HZ, SQ, AK, CXY), and L for low trophic (CW, LHK, NS, XY, SY).

The Han River, the principal affluent of the Yangtze River, is positioned between 106° and 114° east longitude and 30° to 34° north latitude. This river system is a vital component of the Middle Route Project under the South–North Water Transfer Initiative, with its water source originating from the Danjiangkou Reservoir in the central reaches of the Yangtze River. The basin spans approximately 170,400 km^2^ and stretches over roughly 1,570 km. The study focused on distinct segments of the river: upper sections (HZ, NQ, CXY, AK, SQ), central sections (YC, LHK, CW, XY, NS), and lower sections (ZY, QJ, XT, SY, HC). For geographical coordinates and additional details, refer to the Supplementary material (Supplementary Table S1; Figure 1).

### 2.2 Sampling and sample processing

In July 2023, water samples were gathered from multiple layers using a Plexiglas sampler. Samples were collected at 0.5 m below the surface at each site. These samples were then combined and transferred into sterile containers. At each sampling location, five such containers were filled. For phytoplankton analysis, samples were preserved with 1% Lugol’s solution and kept in a portable refrigerator at temperatures below 4 °C. Meanwhile, samples intended for physicochemical analysis and microbial sequencing were stored at −20 °C and subsequently transported to the laboratory. Unfiltered water was utilized for determining TN and TP concentrations, whereas water filtered through 0.45-μm membranes was employed for other indicators. Samples designated for Chl-a analysis were filtered using GF-C membranes and extracted by soaking in 90% acetone for 24 h. Lastly, samples for microbial analysis were filtered through a 0.22 μm filter and stored at −80 °C for DNA extraction and Illumina MiSeq sequencing.

### 2.3 Determination of water environmental parameters

A multiparameter water quality analyzer, such as the YSI Professional Plus, was employed to evaluate water environmental parameters. The determination of TN involved alkaline potassium persulfate digestion. Dissolved inorganic nitrogen (DIN) was quantified, encompassing ammonia nitrogen (NH4 + -N) and nitrate nitrogen (NO3–N). For TP and soluble reactive phosphorus (SRP), the ammonium molybdate method was used. Reagents, digestion times and wavelengths followed Standard Methods 4500-P and 4500-N (American Public Health Association (APHA), 2017). Chemical oxygen demand (COD(Mn)) was assessed via the permanganate index method. In the chlorophyll analysis, samples were filtered using 47 mm diameter GF/C glass fiber filters. Samples were extracted in 90% acetone at 4 °C in the dark for 24 h (Strickland and Parsons, 1972); absorbances were measured at 663 nm and 750 nm and concentrations calculated using SCOR-UNESCO equations.

### 2.4 Detection of phytoplankton and bacteria

#### 2.4.1 Phytoplankton analysis

Following a 48-h sedimentation period, the supernatant from phytoplankton samples was removed, and the volume was adjusted to 50 mL. Identification and enumeration of phytoplankton were performed using a light microscope (Olympus-BX43, Tokyo, Japan). To reduce observer bias, the same analyst (J. Fang, >8 yr. experience) enumerated ≥400 settling units per sample until CV < 10% across 100-unit increments; doubtful taxa were cross-checked at 1000 × oil immersion using Hu and Wei (2006). Morphological criteria for each genus are listed in Supplementary Table S2. Cell counts were conducted in a 0.1-mL plankton counting chamber, with 100 fields examined at 40x magnification. The area per field of view was calculated as S = 0.24 mm^2^. Species richness (S) was determined by counting the number of taxa present. The Shannon diversity (H) index was calculated using the formula (Hill et al., 2003): H=−∑(Pi∗log(Pi)) where denotes the proportional presence of species i within a sample, and S indicates species richness.

#### 2.4.2 Microbial community analysis

Genomic DNA was extracted from water samples using the E. Z. N. A.® Water DNA Kit (OMEGA) according to the manufacturer’s instructions. DNA integrity was confirmed by 1% agarose gel electrophoresis, while DNA concentration and purity were measured using a NanoDrop 2000 UV–vis spectrophotometer (Thermo Scientific, Wilmington, United States). The hypervariable V3-V4 region of the bacterial 16S rRNA gene was amplified using the primer set 338\u00B0F (5′-ACTCCTACGGGAGGCAGCAG-3′) and 806 R (5′-GGACTACHVGGGTWTCTAAT-3′) on an ABI GeneAmp® 9700 PCR system (ABI, CA, United States). PCR products were excised from a 2% agarose gel, purified with the AxyPrep DNA Gel Extraction Kit (Axygen Biosciences, Union City, CA, United States), and quantified using a Quantus™ Fluorometer (Promega, United States).

### 2.5 Calculation of trophic state index

The trophic state of sampling sites was evaluated using the Trophic State Index (TSI). The TSI was determined using a modified version of Carlson’s TSI, considering TN, TP, Chl-a, and Secchi Disk depth (SD) (Zhang et al., 2018). Individual TSI values for each parameter and their composite weighted TSI were calculated using the following equations:


TSI(Chl−a)=10×(2.5+1.086×lnChl−a) (1)

TSI(TP)=10×(9.436+1.624×lnTP) (2)

TSI(TN)=10×(5.453+1.694×lnTN) (3)

TSI(SD)=10×(5.118–1.94×lnSD) (4)

$$\begin{array}{c} \mathrm{TSI}=0.326\times \mathrm{TSI}(\mathrm{Chl}-a)+0.219\times \mathrm{TSI}(\mathrm{TN})\\ +0.230\times \mathrm{TSI}(\mathrm{TP})+0.225\times \mathrm{TSI}(\mathrm{SD}) \end{array}$$ (5)

The units for TN, TP, Chl-a, and SD are milligrams per liter (mg/L), milligrams per liter (mg/L), micrograms per liter (μg/L), and meters (m), respectively. The weights (0.326 Chl-a, 0.219 TN, 0.230 TP, 0.225 SD) follow Zhang et al. (2018), calibrated for Chinese rivers to reduce TP bias. Trophic status classification thresholds based on TSI values are: oligotrophic, TSI < 30; mesotrophic, 30 ≤ TSI ≤ 50; slightly eutrophic, 50 < TSI ≤ 60; moderately eutrophic, 60 < TSI ≤ 70; and hyper-eutrophic, TSI > 70.

### 2.6 Data analysis

Exact sequence variant (ASVs) were generated with UNOISE3 in USEARCH v11; downstream analyses used QIIME 1.9.1. The resulting ASV table was imported into R (v4.3.1) and analyzed using the phyloseq package for alpha- and beta-diversity calculations. The full command-line workflow is provided in Supplementary file S1. Raw sequencing reads were quality-filtered using fastp (version 0.20.0). Sequencing reads were assembled and processed to form a single sequence file after barcode and primer removal using QIIME (version 1.9.1). A total of 7,634,673 high-quality sequences were obtained, with an average depth of 500,231,223 bp for tested samples. All ASV tables were rarefied to 27,000 reads per sample prior to alpha-diversity calculation. Richness and diversity indices, along with redundancy analysis (RDA), were calculated and plotted using R packages “vegan” and “ggplot2.” Relationships between phytoplankton and bacterial communities and environmental variables were analyzed using RDA and VPA. Venn diagrams were generated with the Venn Diagram package in R. Partial Mantel tests assessed correlations between community composition and environmental factors using the “ggcor” package. Spearman correlations were calculated for phytoplankton groups and the most abundant ASVs, as determined by R software. Statistical analyses were conducted using one-way ANOVA with Tukey’s post-hoc test. Co-occurrence networks were constructed using the ‘Psych’ package in R, considering ASVs with a relative abundance above 0.01%. Edges retained only when Spearman |*r*| ≥ 0.6 and FDR-adjusted *p* < 0.05 (Fang et al., 2017). Network visualization was facilitated by Gephi software. Additional data analyses were performed in R (version 4.3.1).

## 3 Results

### 3.1 Water chemistry and trophic state of the Han River Basin

Most sampling sites (75%) were eutrophic, with TSI values ranging from 42.5 to 66.0 and a median TSI of 55.5, and the results are derived using the methods described in Equations 1–5. According to trophic state classification criteria, there were 7 mesotrophic sites, 14 slightly eutrophic sites, and 7 moderately eutrophic sites. TN, TP, and Chl-a concentrations varied from 1.10 to 6.00 mg/L, 0.010 to 0.29 mg/L, and 0.86 to 70.00 μg/L, respectively. Average concentrations were 2.90 ± 0.91 mg/L for TN, 0.09 ± 0.06 mg/L for TP, and 6.30 ± 6.30 μg/L for Chl-a (Table 1; Supplementary Table S5).

**Table 1:** Descriptive statistics of TN, TP, Chl*-*a, SD, and TSI.

	TN (mg/L)	TP (mg/L)	Chl-a (μg/L)	SD (m)	TSI
Min	1.10	0.01	0.86	0.10	42.50
Max	6.00	0.29	70.00	5.00	66.00
Median	2.70	0.08	3.10	0.80	55.50
Mean	2.90	0.09	6.30	0.79	55.60
Standard deviation	0.97	0.06	6.30	0.63	7.30

### 3.2 Composition and distribution of bacterial and phytoplankton communities

**Figure 2:**
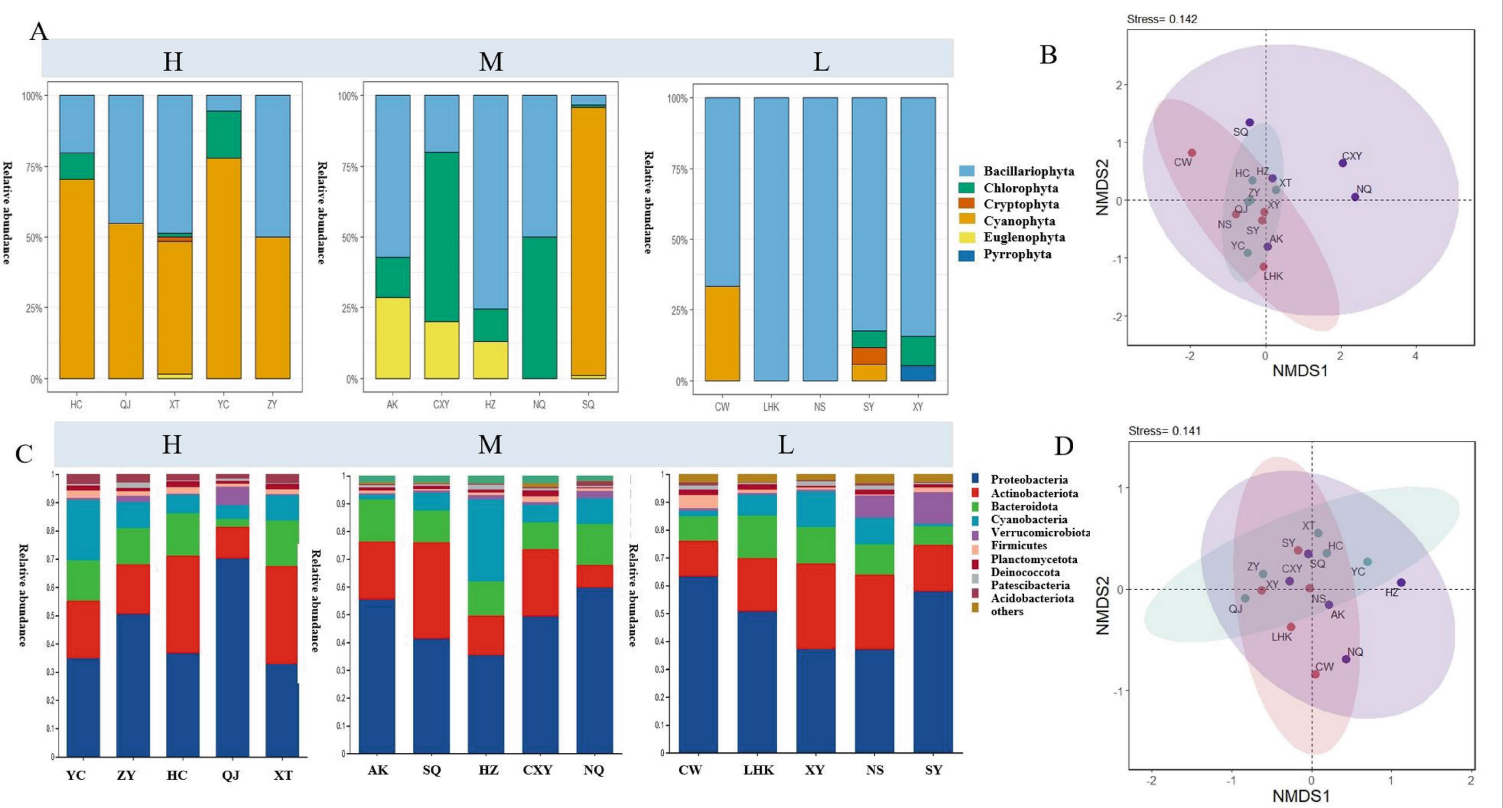
Variations in the relative abundance of phytoplankton **(A)** and bacteria **(C)** at the phylum level under different eutrophication levels in the Han River Basin. Non-metric multidimensional scaling (NMDS) plots of phytoplankton **(B)** and bacterial **(D)** genera based on Bray-Curtis dissimilarities in taxonomic compositions among all samples [low trophic (L), medium trophic (M), and high trophic (H)]. The proximity of points on the plot indicates the degree of community similarity at the genus level.

A total of 6 phyla and 33 genera of phytoplankton were identified in all samples within the study area (Supplementary Table S3). The distribution and relative abundance of different phyla of phytoplankton from different eutrophication levels are shown in Figure 2A. These six phyla are *Cyanophyta, Chlorophyta, Pyrrophyta, Bacillariophyta, Cryptophyta*, and *Euglenophyta*. The *Bacillariophyta* includes 11 genera (accounting for 33.33% of the total species count). The most abundant species are the genus *Mallomonas* and the genus *Chlamydomonas*, representing 21.80 and 13.69% of the total phytoplankton cell density, respectively. The *Bacillariophyta* shows a clear dominance of a single species in the phytoplankton community in areas of relatively low trophic levels (L, 30 ≤ TSI ≤ 50). In areas of relatively high trophic levels (H, 60 < TSI ≤ 70), the *Cyanophyta* exhibits a clear dominance of a single species. The *Cyanophyta* includes seven species (accounting for 21.21% of the total species count). The most abundant genus are the *Microcystis* and the *Merismopedia*, representing 22.29 and 12.71% of the total phytoplankton cell density, respectively. Non-metric multidimensional scaling (NMDS) analysis indicates that differences in trophic levels have stratified the phytoplankton communities according to different sampling areas (Figure 2B). Among the three differentiated regions, the region with a relatively medium trophic level (M, 50 < TSI ≤ 60) has the highest phytoplankton cell density (8.18 × 10^8^ cells/L), while the L region has the lowest phytoplankton cell density (1.60 × 10^8^ cells/L) and also the lowest diversity index (Shannon index) of phytoplankton (Supplementary Tables S3, S4). A total of 18 bacterial phyla and 32 classes were identified in this study. As shown in Supplementary Figure S1, there are 203 core species among all samples, with the main composition at the phylum level being *Proteobacteria*. Like phytoplankton, bacterial community composition also varies with eutrophication levels (Figure 2D). However, bacterial communities change more on a spatial scale (Figure 2C). On a spatial scale, the relative abundance of *Proteobacteria* in QJ and CW is 70.18 and 63.28%, respectively, which is different from other locations (Figure 2C). Notably, the proportion of *Cyanobacteria* at the HZ site is the highest (29.59%). At the genus level, *Acinetobacter* is significantly more abundant in the QJ and LHK areas, while the hgcI_clade branch is more abundant in the XT and SQ areas (Supplementary Figure S2). The diversity index (Shannon index) shows significant differences on a spatial scale between the L and H areas (Supplementary Table S4). The bacterial Shannon index at the QJ site is significantly lower than other sites in the same area (*p* < 0.05). In addition, bacterial community diversity also shows differences with eutrophication levels. The average value of the diversity index in the L area is significantly higher than in the other two areas (*p* < 0.05), and the higher the eutrophication level, the lower the Shannon index (Supplementary Table S4).

### 3.3 The impact of pollution on bacterial and phytoplankton communities

**Figure 3:**
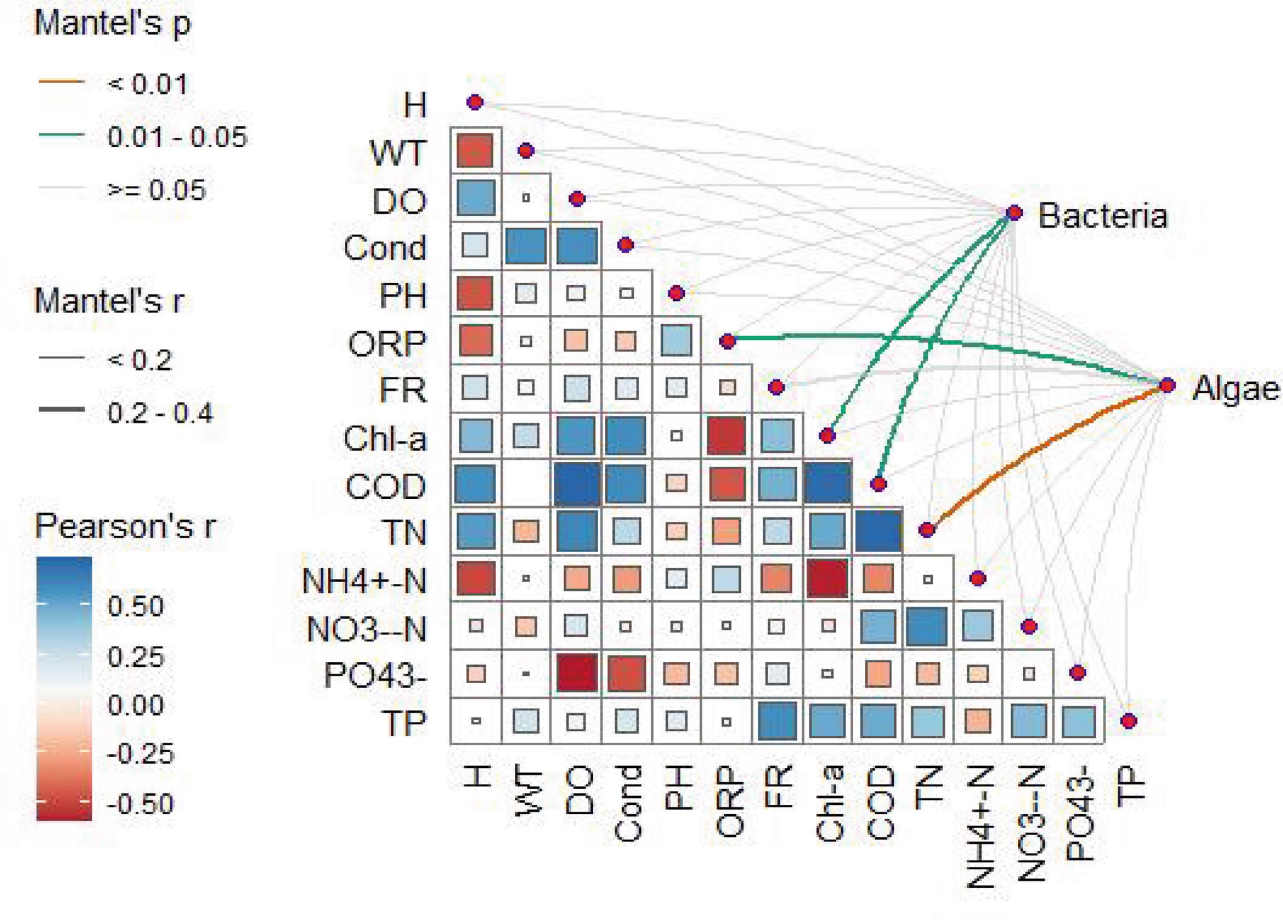
Mantel test-based analysis of the relationship between environmental variables and the phytoplankton and bacterial communities classified at the phylum level. H, Secchi Disk depth; WT, Water; Temperature; TN, total nitrogen; NH_4_^+^-N, Ammonium Nitrogen; NO₃^−^-N, Nitrate Nitrogen; PO₄^3−^, Orthophosphate; TP, total phosphorus; Chl-a, chlorophyll-a; COD, chemical oxygen demand; FR, Flow Velocity; DO, dissolved oxygen; Cond, conductivity; ORP, oxidation–reduction potential.

To investigate the correlation between planktonic microbial communities and environmental variables, a Monte Carlo test analysis was conducted for all environmental factors and community matrices at the phylum level (Figure 3; Supplementary Table S6). Many environmental variables correlated with species, but the distribution patterns of correlations differed between phytoplankton and bacterial communities. Variables most closely associated with bacterial communities were chl-a and COD (correlation coefficients of 0.30 and 0.29, respectively, 0.01 < *p* < 0.05). Phytoplankton communities primarily correlated positively with certain nutrients, such as TN (correlation coefficient of 0.39, *p* < 0.01).

**Figure 4:**
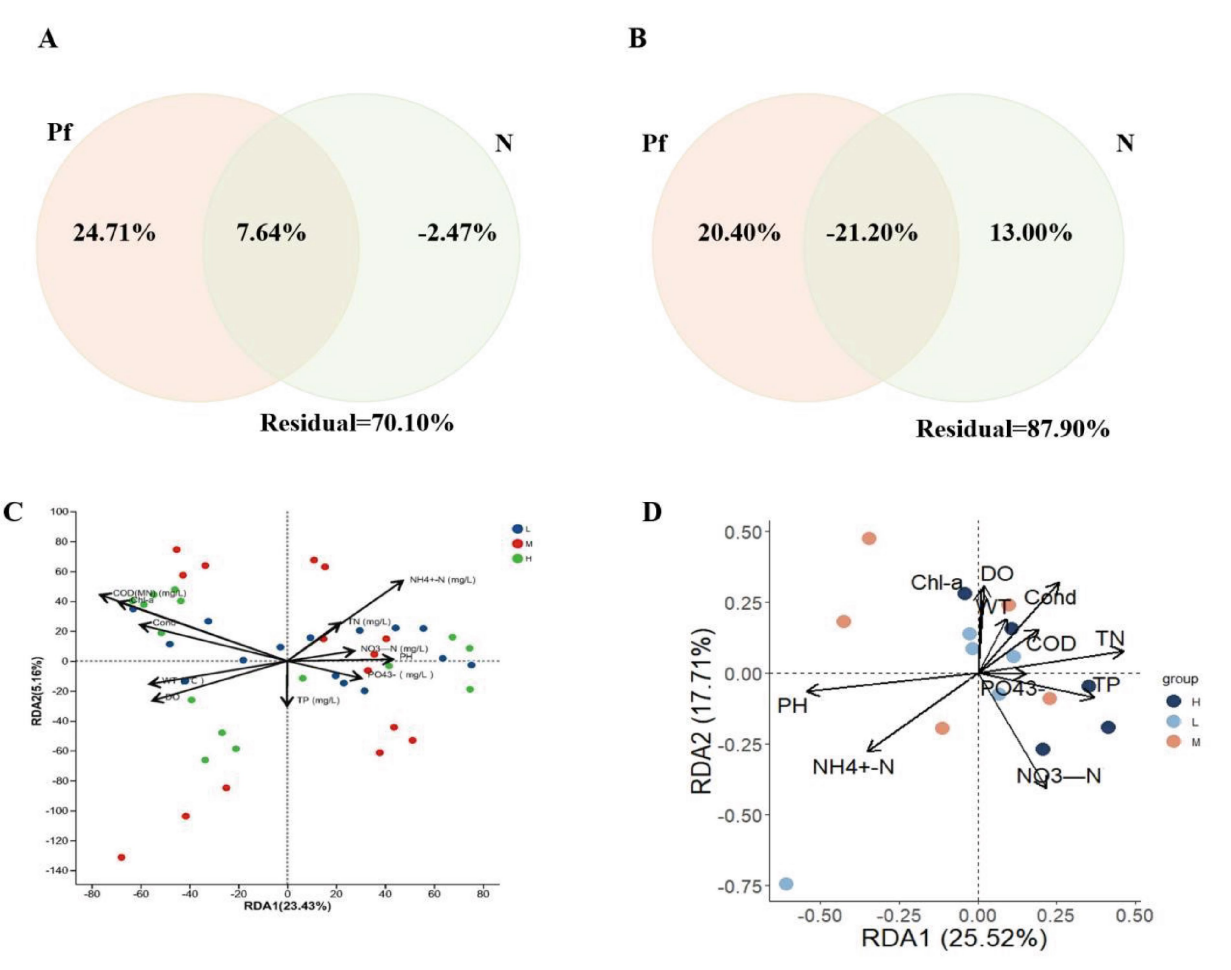
Driving factors of phytoplankton and bacterial communities in the Han River Basin. Variation partitioning analysis (VPA) illustrating the contribution of environmental factors to the variance in bacterial **(A)** and phytoplankton **(B)** communities (*p* < 0.05). Negative unique fractions are mathematical artifacts that occur when the covariance between predictor sets exceeds their individual unique contributions. The analysis of physical factors included water temperature (°C), dissolved oxygen (DO), conductivity (Cond), pH, and chlorophyll a (Chl-a). The analysis of nutrients included total nitrogen (TN), nitrate nitrogen (NO^3^-N), ammonium nitrogen (NH_4_^+^-N), total phosphorus (TP), phosphate (PO_4_^3^-), and chemical oxygen demand (COD). Redundancy analysis (RDA) between environmental variables and bacterial **(C)** and phytoplankton **(D)** communities (Analysis of Variance, ANOVA, *p* < 0.01).

Redundancy analysis (RDA) and variation partitioning analysis (VPA) were performed to identify the main environmental factors affecting the planktonic microbial communities. VPA results indicated that environmental factors explained a greater proportion of the variation in bacterial community composition (29.90%) than in phytoplankton communities (12.10%) (Figures 4A,B). When all environmental factors were divided into two groups, physical factors had a more significant impact on bacterial communities (32.36%) compared to nutrient variables (11.93%) (Figure 4B). This confirmed the results of the Monte Carlo test. Subsequently, RDA was conducted on the selected environmental variables and phytoplankton and bacterial communities (Figures 4C,D). Results showed that these environmental variables accounted for 43.23% of the variation in phytoplankton communities (RDA1 = 25.52%, RDA2 = 17.71%) (Figure 4C) and 28.59% of the variation in bacterial communities (RDA1 = 23.43%, RDA2 = 5.16%) (Figure 4D). Phytoplankton communities were more distinctly separated along the eutrophication gradient and clustered according to different sampling areas, while bacterial communities were primarily influenced by physical factors.

### 3.4 The impact of eutrophication on bacterial-phytoplankton communities

**Figure 5:**
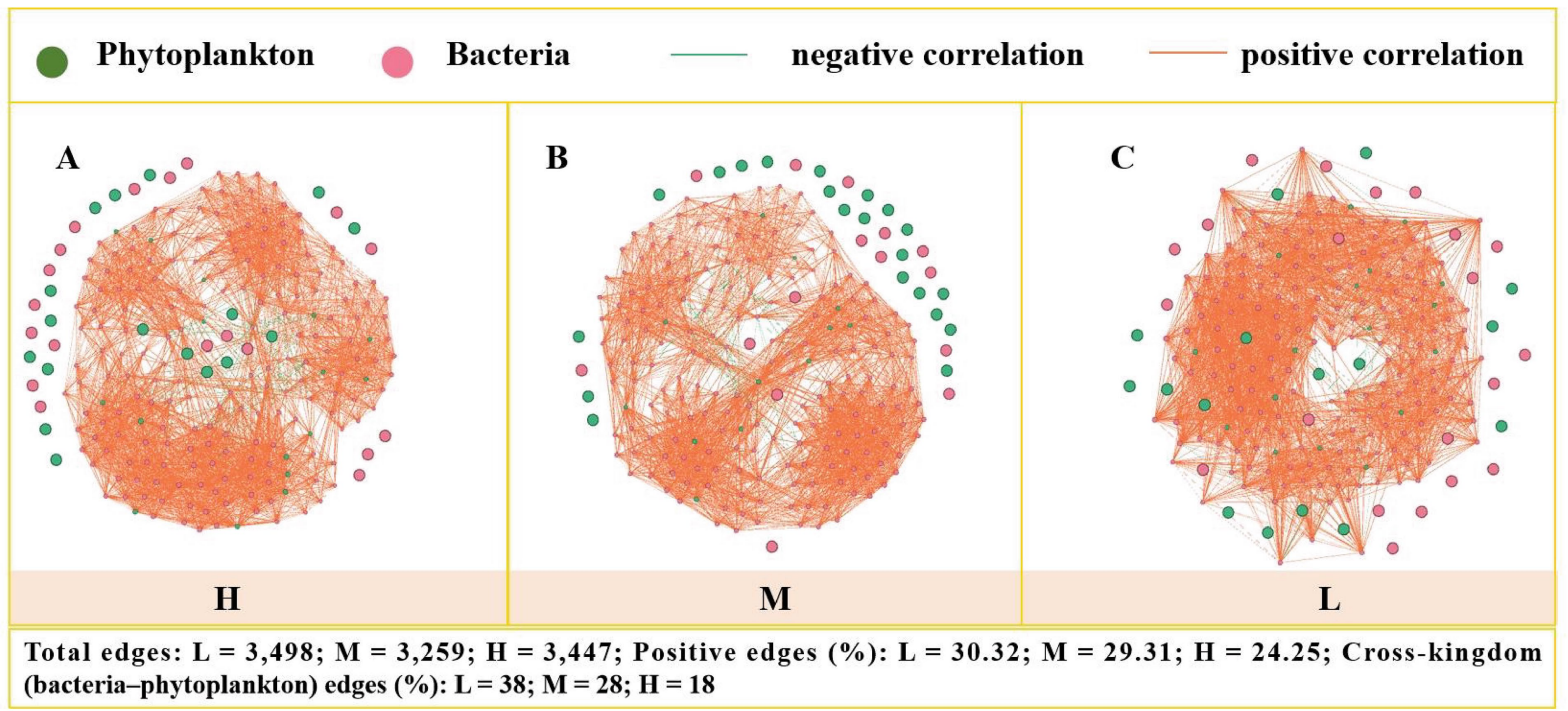
Co-occurrence networks of phytoplankton (green nodes) and bacterial (red nodes) ASVs across low **(A, L)**, medium **(B, M)**, and high **(C, H)** trophic gradients. Node color: green represents phytoplankton, red represents bacteria. Edge color: red indicates a positive correlation (*r* > 0.6, *p* < 0.05), green indicates a negative correlation (*r* < -0.6, *p* < 0.05). The networks include three pairwise interaction types: bacteria–bacteria, phytoplankton–phytoplankton, and bacteria–phytoplankton.

Separate co-occurrence networks of phytoplankton and bacteria were generated for each gradient under different levels of eutrophication to suggest their association patterns across different scales (Figure 5). Topological properties of all networks were calculated to distinguish changes in co-occurrence networks under varying gradients of eutrophication (Table 2). Network topological clustering coefficients, modularity indices, and the percentages of positive and negative correlations were used to assess changes in connections within phytoplankton and bacterial communities. In areas with low levels of eutrophication, the highest modularity index of 0.641 was observed, indicating that phytoplankton and bacteria each formed relatively independent community modules. As pollution levels increased from moderate to high, the modularity index decreased from 0.598 to 0.558, showing that the independent modules of different species were gradually disrupted, and connections between phytoplankton and bacteria became more intimate. Within co-occurrence networks, as eutrophication levels increased, phytoplankton and bacteria moved closer and connected more frequently. In heavily polluted areas, the clustering coefficient reached its peak, indicating that organisms in this environment formed highly connected groups. The average path length decreased from 2.337 in the L area to 2.26 in the H area, and the diameter also decreased from 5 in the L area to 4 in the H area, further indicating that as pollution levels increased, the networks became more compact and efficient. Regarding the percentages of positive and negative correlations, as eutrophication levels increased, the percentage of negative correlations also increased, from 0.32% in the L area to 0.61% in the H area (Table 2), which may reflect that interspecies competition and predation relationships become more pronounced at higher pollution levels. Overall, these results suggest that as eutrophication levels increase, the co-occurrence networks of phytoplankton and bacteria become more tightly knit, and interactions between species become more complex. These changes may have significant implications for ecosystem functionality and stability.

**Table 2:** The topological characteristics of phytoplankton and bacterial co-occurrence networks in different groups.

	L	M	H
Node	233	233	233
Edge	3,498	3,259	3,447
Average degree	30.026	27.974	29.588
Diameter	5	4	4
Clustering coefficient	0.719	0.678	0.682
Modularity index	0.641	0.598	0.558
Average path length	2.337	2.369	2.26
Positive	30.32	29.31	24.25
Negative	0.32	0.36	0.61

With increasing trophic state, total network edges remained relatively stable but modularity declined (0.641 → 0.558), indicating collapsing module boundaries. Most notably, the proportion of positive cross-kingdom (bacteria–phytoplankton) edges decreased from 38% in L to 18% in H, while negative correlations rose from 0.32 to 0.61%. This suggests that eutrophication weakens mutualistic interactions and intensifies competition or antagonism between phytoplankton and bacteria.

## 4 Discussion

### 4.1 General characteristics of water quality and trophic status in the Han River Basin

Water quality monitoring results reveal the ubiquity and severity of eutrophication issues in the Han River Basin, with 75% of sampling sites classified as eutrophic, exhibiting TSI values ranging from 42.5 to 66.0, and a median TSI of 55.5 (Table 1). This indicates a gradient of trophic states from oligotrophic to eutrophic conditions across the basin, with most waters impacted by nutrient pollution (Zhuo et al., 2023). TN, TP, and Chl-a concentrations show significant variability, with mean values of 2.90 ± 0.91 mg/L, 0.09 ± 0.06 mg/L, and 6.30 ± 6.30 μg/L (Table 1), respectively, confirming widespread nutrient pollution in the Han River Basin (Zhuo et al., 2023).

Water quality issues in the Han River Basin may be attributed to agricultural activities and urbanization processes within the basin. The extensive use of chemical fertilizers and pesticides in agricultural practices, along with urbanization-related factors such as surface hardening, wastewater discharge, and garbage accumulation, can lead to nutrient accumulation and promote eutrophication (Zhang et al., 2021). Moreover, spatial heterogeneity in trophic status across the Han River Basin may be associated with varying land-use patterns, distribution of pollution sources, and the self-purification capacity of water bodies (Li et al., 2018).

Water quality and trophic status in the Han River Basin have significant implications for ecosystem health and stability. Eutrophication can alter the structure of algal and bacterial communities in water bodies, potentially affecting the entire ecosystem’s functionality and stability through the food web (Zhang et al., 2021). Therefore, water quality management and ecological conservation in the Han River Basin require comprehensive consideration of various pollution sources and environmental factors within the basin. Effective measures must be implemented to reduce nutrient inputs and restore the health of aquatic ecosystems (Zhuo et al., 2023). This includes improving agricultural practices to minimize nutrient runoff, enhancing wastewater treatment to reduce pollutant discharge, and promoting urban planning strategies that incorporate green spaces and wetlands to enhance nutrient adsorption and degradation.

### 4.2 Impacts of eutrophication on phytoplankton and bacterial communities

The impacts of eutrophication on phytoplankton and bacterial communities are complex. This study found that diatoms dominated in low-nutrient areas, while cyanobacteria became the dominant group in high-nutrient areas (Figure 2). This shift may be related to the adaptability of different algae to nutrient conditions. In relatively low-nutrient environments, diatoms dominate due to their efficient nutrient utilization. Diatoms can maintain high growth rates under low nutrient concentrations through complex photosynthetic mechanisms (Zhou et al., 2021). However, when nutrient concentrations in water bodies increase, the competitive advantage of cyanobacteria becomes apparent. The increase in cyanobacteria may be related to their competitive edge under high-nutrient conditions, consistent with previous research (Burford et al., 2020). Some cyanobacteria species, such as Microcystis and Anabaena, can rapidly reproduce in high-nutrient environments, leading to algal blooms (Song et al., 2023). These cyanobacteria can tolerate high nutrient concentrations and quickly deplete nutrients in water through photosynthesis, restricting the growth of other algae. Additionally, cyanobacteria can produce compounds resistant to grazing and pathogens, further enhancing their competitiveness in high-nutrient environments (Song et al., 2023). Integrating 18S rRNA or ITS gene sequencing with the current microscopic counts will help clarify whether the observed shift from diatoms to cyanobacteria is accompanied by an increase in overlooked picoeukaryotes that could influence nutrient cycling and food-web dynamics.

Bacterial communities exhibited significant spatial heterogeneity, with *Proteobacteria* dominating across all sites (mean relative abundance >60%, especially in QJ and LHK), consistent with their versatile metabolic capabilities in nutrient-rich environments (Huber et al., 2020). Within *Proteobacteria*, the genus *Acinetobacter* was notably enriched in high-trophic sites (e.g., QJ, LHK), suggesting its role in organic matter decomposition and potential pathogenicity under eutrophic conditions. Conversely, the hgcI_clade (*Actinobacteria*) showed higher relative abundance in low-trophic regions (e.g., SQ, XT), indicating its preference for oligotrophic conditions and potential involvement in recalcitrant organic matter processing. Additionally, Cyanobacteria, primarily Microcystis-associated taxa, increased significantly in high-trophic sites (e.g., HZ), aligning with their capacity to exploit high nitrogen/phosphorus levels and form harmful algal blooms (Song et al., 2023). These taxon-specific responses underscore the functional turnover of bacterial communities along the eutrophication gradient, with implications for nutrient cycling (e.g., denitrification by *Proteobacteria*) and ecosystem stability. While temperature and pH varied across sampling sites, they were not identified as the primary drivers of bacterial community structure in this study (Mantel test, *p* > 0.05). Instead, Chl-a and COD emerged as the most significant explanatory variables (Figure 3), suggesting that organic-matter availability and phytoplankton-derived substrates exert stronger control over bacterial assemblages than broad physicochemical gradients. These environmental factors collectively act on bacterial communities, leading to dynamic changes in community structure and function, which in turn affect nutrient cycling and biogeochemical processes in water bodies (Huber et al., 2020). Therefore, understanding the impacts of eutrophication on phytoplankton and bacterial communities is crucial for developing effective water management strategies. This may involve monitoring and controlling nutrient inputs and taking measures to restore the natural purification capacity of water bodies.

We acknowledge a discrepancy in the relative abundance of Cyanobacteria between Figure 2A (microscopy-based phytoplankton identification) and Figure 2C (16S rRNA gene sequencing). Specifically, several samples with undetectable cyanobacterial cells under microscopy showed non-negligible proportions of cyanobacterial reads in the sequencing data. This inconsistency likely arises from methodological differences: microscopy may underestimate small or morphologically ambiguous cyanobacteria (e.g., *picocyanobacteria*) (Komárek, 2016), while high-throughput sequencing can detect extracellular DNA, dead cells, or even plastid-derived sequences misclassified as cyanobacteria (Huse et al., 2007). These limitations highlight the complementary yet non-overlapping nature of morphological and molecular approaches, and caution against direct quantitative comparison between the two.

### 4.3 Impacts of eutrophication on phytoplankton-bacterial community networks

The impacts of eutrophication on phytoplankton-bacterial community networks are multi-dimensional, involving ecological interactions, community structure changes, and dynamic adjustments in biogeochemical cycles. Results show that as eutrophication levels increase, the co-occurrence networks of phytoplankton and bacterial communities become more tightly interconnected (Figure 5; Table 2). This may be related to the environmental filtering effect caused by eutrophication, where certain species with strong tolerance dominate under high-nutrient conditions, reducing community diversity and complexity (Machado et al., 2023). For example, cyanobacteria reproduce rapidly in eutrophic environments, forming blooms that inhibit the growth of other phytoplankton, leading to a decrease in overall community diversity (Zuo et al., 2024).

Such changes in community structure not only affect interactions between phytoplankton and bacteria but may also have profound effects on the biogeochemical cycles of aquatic bodies (Lobus and Kulikovskiy, 2023). As primary producers in aquatic bodies, phytoplankton’s photosynthetic activities directly affect carbon fixation and organic matter production. Under eutrophication conditions, certain rapidly reproducing phytoplankton species may dominate, leading to a reduction in other species. This changes the quality and availability of organic matter and may affect the structure of the food web and the efficiency of energy flow. For instance, cyanobacterial blooms may produce toxins that inhibit the growth of other phytoplankton and affect zooplankton and fish that feed on phytoplankton (Song et al., 2023). The role of bacteria in decomposing organic matter and transforming nutrients is also affected by changes in community structure. In eutrophic environments, bacterial communities may shift toward species that can utilize high concentrations of nutrients, participating more effectively in denitrification and nitrogen fixation, thus affecting the nitrogen cycle. However, this change may reduce the overall efficiency of nutrient cycling, as some bacteria may focus more on competitively absorbing nutrients rather than promoting their cycling (Hutchins and Capone, 2022). This change may lead to nutrient accumulation in water bodies, further exacerbating eutrophication issues.

The increase in negative correlations indicates that under eutrophication conditions, interspecies competition and predation relationships become more significant (Li et al., 2022). The intensification of these relationships may be due to excessive competition for resources, especially when resources such as nutrients and light are limited. Increased competition may lead to a decline in the population of certain species, and the enhancement of predation relationships may affect the stability of the food web (Xu et al., 2023). For example, certain bacteria may enhance their competitiveness by producing compounds that inhibit the growth of other bacteria or phytoplankton, and the production of these chemical substances may change the composition and function of the community (Xu et al., 2023). Additionally, the rapid growth of phytoplankton may lead to intensified competition for bacterial resources, as bacteria need to decompose the organic matter produced by phytoplankton (Reinl et al., 2022). In this case, bacterial communities may suppress the growth of phytoplankton through selective predation or competition, forming complex ecological dynamics. This dynamic may affect the population structure of phytoplankton and, in turn, affect the primary productivity and stability of the food web in water bodies. For example, certain bacteria may specifically prey on certain phytoplankton, thereby reducing their numbers and increasing opportunities for other phytoplankton, leading to further changes in community structure (Costas-Selas et al., 2023).

Therefore, the impact of eutrophication on phytoplankton-bacterial community networks is not only related to changes in community structure but may also have profound effects on the function and stability of the entire ecosystem. This emphasizes the need to consider the relationship between community structure and function in eutrophication management and how to improve ecosystem resilience by restoring ecological balance. For example, reducing nutrient input and restoring wetlands and vegetation cover can promote diversity recovery, thereby enhancing the stability and resistance of ecosystems. Future research needs to further explore the specific impacts of eutrophication on ecosystem functions and how to restore damaged ecosystems through ecological restoration. Special attention should be paid to the impact of different nutrient concentrations on the interactions between phytoplankton and bacterial communities to provide a scientific basis for water management and ecological restoration. At the same time, exploring how to use the advantages of phytoplankton-bacterial symbiotic networks to develop new water management and pollution remediation technologies will be an important direction for future research.

### 4.4 Limitations

Because this study is observational, causal mechanisms (e.g., toxin production, nutrient uptake kinetics) remain to be validated under controlled conditions. Future mesocosm experiments and functional-gene assays are recommended to link community shifts to ecosystem processes. Additionally, the observed discrepancy between microscopy and sequencing in detecting cyanobacteria underscores the need for cautious interpretation of taxonomic abundance, especially when integrating datasets from different analytical platforms.

## 5 Conclusion

This study provides insights into the ecological consequences of eutrophication on phytoplankton and bacterial communities in the Han River Basin, a critical water source for the Middle Route Project of the South-to-North Water Diversion in China. Findings indicate that 75% of sampling sites are eutrophic, with significant fluctuations in TN, TP, and Chl-a concentrations, reflecting widespread nutrient pollution. The shift in phytoplankton dominance from diatoms to cyanobacteria with increasing eutrophication levels suggests adaptation to nutrient-rich conditions, potentially leading to algal blooms and toxin production, which can disrupt the food web and ecosystem stability. Bacterial communities also exhibit spatial variability, with *Proteobacteria* being the core species, and their diversity decreases with higher eutrophication levels. Co-occurrence networks reveal a more interconnected community structure under eutrophic conditions, with increased negative correlations suggesting intensified interspecies competition and predation. These changes in community networks could have profound implications for ecosystem functionality and stability. Therefore, effective nutrient management and ecological restoration are crucial to mitigate eutrophication and its adverse effects on the Han River Basin’s ecosystem health. Future research should focus on developing strategies to enhance ecosystem resilience and explore the potential of phytoplankton-bacterial interactions for innovative water management and pollution remediation technologies.

## Data Availability

The raw 16S rRNA gene sequences reported in this study have been deposited in the NCBI Sequence Read Archive under BioProject accession number PRJNA1308029 (https://www.ncbi.nlm.nih.gov/bioproject/PRJNA1308029).
