# Supplementary material for: Ecological responses of phytoplankton and bacterial communities to eutrophication in the Han River Basin
Source: Front Microbiol. 2026 Jan 5;16:1649806. doi: 10.3389/fmicb.2025.1649806 (PMC12812980; doi:10.3389/fmicb.2025.1649806)
Supplement: Supplementary file 1 [file Supplementary_file_1.docx]

**Ecological Responses of Phytoplankton and Bacterial Communities to Eutrophication in the Han River Basin**

Yuanyuan Chen ^1,2^, Fangtao Cai^1^, Zhiyuan Qi^1^, Tianqi He^1^, Jiao Fang^3^, Dongdong Zhai^1,2^, Hongyan Liu^1,2^, Ming Xia^1,2^, Zhangfeng Hu^3^,Yanfu Que^4^, Fei Xiong^1,2^, *Bin Zhu^4^*

1. Hubei Engineering Research Center for Protection and Utilization of Special Biological Resources in the Hanjiang River Basin, School of Life Sciences, Jianghan University, Wuhan, China
2. Institute of Microalgae Synthetic Biology and Green Manufacturing, School of Life Sciences, Jianghan University, Wuhan , China.
3. Hubei Key Laboratory of Environmental and Health Effects of Persistent Toxic Substances, Jianghan University, Wuhan, China
4. Institute of Hydroecology, Ministry of Water Resources and Chinese Academy of Sciences, Wuhan, China

*Correspondence: Fei Xiong. School of Life Sciences, Jianghan University, Wuhan 430056, China. E-mail: [xf9603@163.com](mailto:xf9603@163.com); Bin Zhu. Institute of Hydroecology, Ministry of Water Resources and Chinese Academy of Sciences, Wuhan430079, China. E-mail:zhubin@mail.ihe.ac.cn.

**Supplemental figures and tables**

**Figure S1
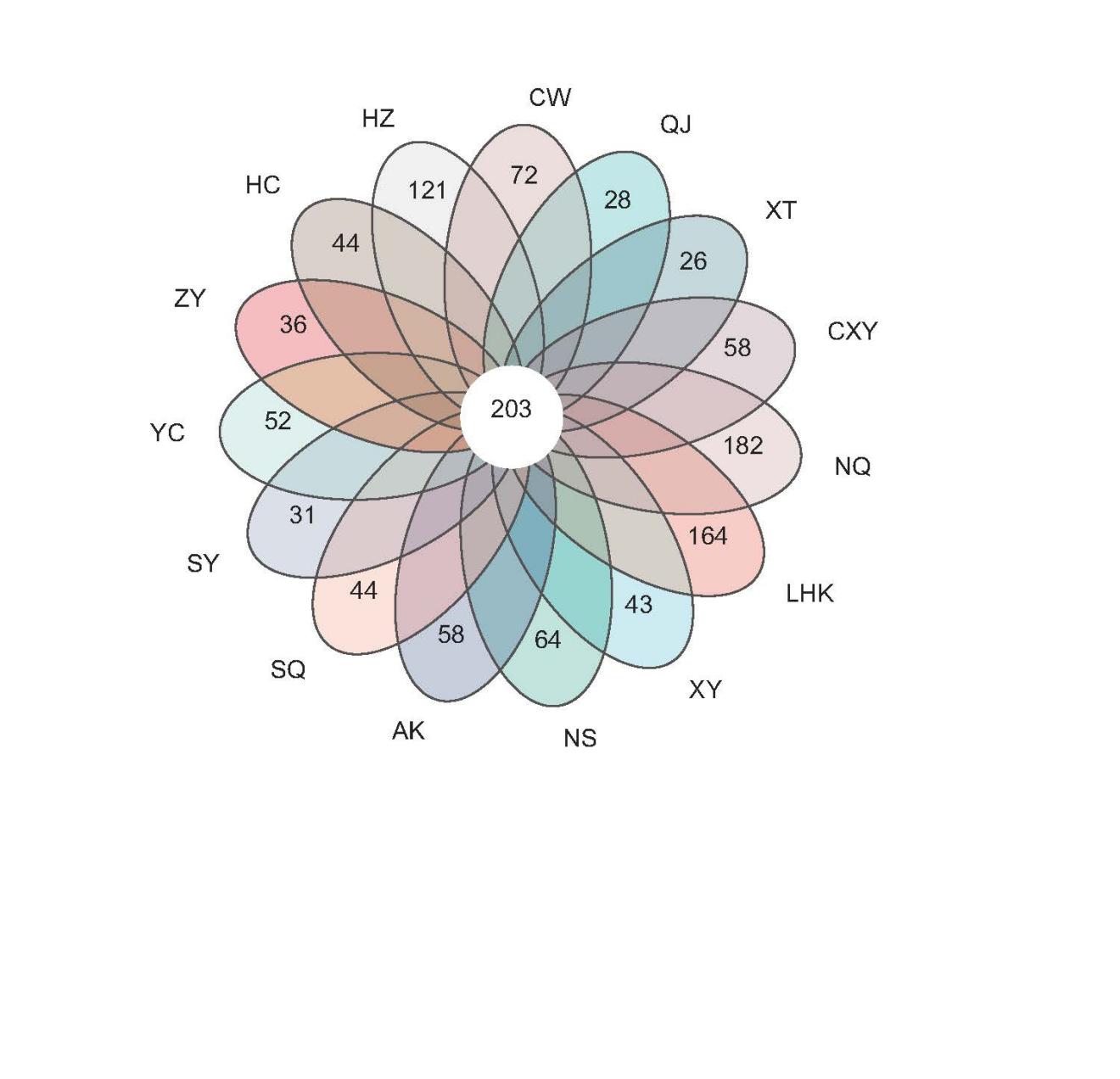
 Venn diagram of water bacterial ASVs across all sampling sites.**


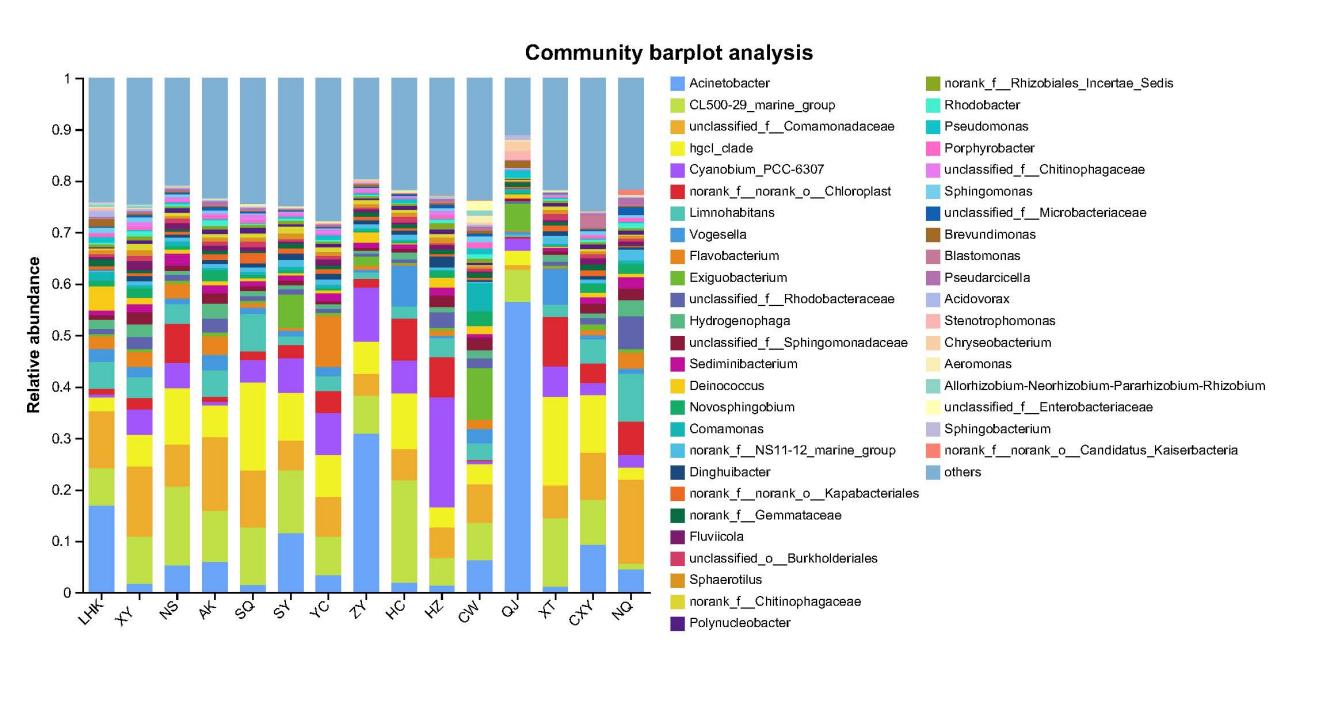


**Figure S2 Bacterial community composition with relative abundance>5% at the genus level.**

| **Table S1 Geographical locations (coordinates) of sampling sites** | | | |
| --- | --- | --- | --- |
| Site | East Longitude | Northern Latitude | Trophic Group |
| AK_1 | 109.00.46.97 | 32.42.03.52 | M |
| AK_2 | 109.72.64.52 | 32.54.69.16 | M |
| AK_3 | 107.69.43.09 | 33.01.73.72 | M |
| CXY_1 | 109.23.38.42 | 32.49.53.88 | M |
| CXY_2 | 109.25.46.32 | 32.44.53.79 | M |
| CXY_3 | 109.01.06.41 | 32.42.04.63 | M |
| HZ_1 | 106.55.45.39 | 33.06.13.35 | M |
| HZ_2 | 106.53.54.09 | 33.06.33.39 | M |
| HZ_3 | 106.51.69.02 | 33.06.31.54 | M |
| NQ_1 | 106.14.41.57 | 32.49.19.29 | M |
| NQ_2 | 106.14.40.77 | 32.49.19.85 | M |
| NQ_3 | 106.14.40.28 | 32.49.20.46 | M |
| SQ_1 | 108.14.29.54 | 33.02.19.95 | M |
| SQ_2 | 108.14.26.99 | 33.02.11.66 | M |
| SQ_3 | 108.13.24.08 | 33.03.01.69 | M |
| CW_1 | 111.27.49.92 | 32.33.37.85 | L |
| CW_2 | 111.22.56.85 | 32.31.17.56 | L |
| CW_3 | 111.23.15.14 | 32.41.02.58 | L |
| XY_1 | 112.09.26.10 | 32.02.30.95 | L |
| XY_2 | 112.09.32.83 | 32.02.20.45 | L |
| XY_3 | 112.09.41.81 | 32.02.08.03 | L |
| LHK_1 | 111.41.00.42 | 32.17.16.75 | L |
| LHK_2 | 111.41.08.77 | 32.17.19.53 | L |
| LHK_3 | 111.41.20.77 | 32.17.24.88 | L |
| NS_1 | 112.01.06.66 | 32.04.39.68 | L |
| NS_2 | 112.01.08.09 | 32.04.03.98 | L |
| NS_3 | 112.01.10.56 | 32.04.20.61 | L |
| YC_1 | 112.16.45.80 | 31.43.22.43 | H |
| YC_2 | 112.16.45.30 | 31.43.48.87 | H |
| YC_3 | 112.16.40.63 | 31.43.07.90 | H |
| ZY_1 | 112.26.02.42 | 31.28.12.05 | H |
| ZY_2 | 112.26.09.31 | 31.28.15.12 | H |
| ZY_3 | 112.26.24.26 | 31.28.17.96 | H |
| SY_1 | 112.33.10.31 | 30.53.14.08 | L |
| SY_2 | 112.33.21.24 | 30.53.18.74 | L |
| SY_3 | 112.33.16.00 | 30.53.16.97 | L |
| QJ_1 | 112.53.7.12 | 30.29.41.01 | H |
| QJ_2 | 112.48.56.04 | 30.30.36.43 | H |
| QJ_3 | 112.48.57.72 | 30.30.35.09 | H |
| XT_1 | 113.28.26.01 | 30.23.36.80 | H |
| XT_2 | 113.28.18.65 | 30.23.36.03 | H |
| XT_3 | 113.28.01.54 | 30.23.37.95 | H |
| HC_1 | 113.38.53.77 | 30.34.31.28 | H |
| HC_2 | 113.38.53.39 | 30.34.28.48 | H |
| HC_3 | 113.38.54.68 | 30.34.26.11 | H |

| **Table S2 Phytoplankton genera identified by light microscopy, their relative abundances, and taxonomic-morphological criteria used for classification in the Han River Basin.** | | | |
| --- | --- | --- | --- |
| Genus | Main Phylum | Relative Abundance (%) | Diagnostic Characters & Key References |
| Microcystis | Cyanophyta | 22.29 | Spherical colonies 3–7 µm, no aerotopes; GB 17378.7-2007; Wehr et al. 2015 |
| Merismopedia | Cyanophyta | 12.71 | Flat square colonies of 4–8 cells in regular rows; Hu & Wei 2006 |
| Mallomonas | Bacillariophyta | 21.80 | Siliceous scales and apical bristles visible at 1000×; John et al. 2022 |
| Chlamydomonas | Chlorophyta | 13.69 | Oval cells with 2 equal flagella, cup-shaped chloroplast; Wehr et al. 2015 |
| Cyclotella | Bacillariophyta | 9.14 | Discoid valves, radial striae, hyaline central area; Hu & Wei 2006 |
| Aphanizomenon | Cyanophyta | 7.23 | Straight filaments, cylindrical cells, terminal heterocytes; Wehr et al. 2015 |
| Scenedesmus | Chlorophyta | 4.87 | Coenobia of 4–8 linear/curved cells; John et al. 2022 |
| Melosira | Bacillariophyta | 4.12 | Cylindrical frustules forming filaments, distinct girdle bands; Hu & Wei 2006 |
| Cryptomonas | Cryptophyta | 3.98 | Dorsiventrally flattened cells with 2 unequal flagella; Wehr et al. 2015 |
| Peridinium | Pyrrophyta | 3.45 | Large theca with prominent girdle and sulcus; John et al. 2022 |
| Euglena | Euglenophyta | 3.02 | Spindle-shaped cells with 1 emergent flagellum, stigma present; Hu & Wei 2006 |
| Anabaena | Cyanophyta | 2.88 | Filaments with spherical akinetes adjacent to heterocytes; Wehr et al. 2015 |
| Synedra | Bacillariophyta | 2.61 | Needle-shaped frustules, no raphe; John et al. 2022 |
| Pediastrum | Chlorophyta | 2.30 | Flat coenobia with marginal spines; Wehr et al. 2015 |
| Ceratium | Pyrrophyta | 2.15 | Horned cells, 2–3 antapical spines; John et al. 2022 |
| Fragilaria | Bacillariophyta | 1.97 | Rectangular cells in ribbon-like colonies; Hu & Wei 2006 |
| Navicula | Bacillariophyta | 1.82 | Boat-shaped valves, distinct raphe; Wehr et al. 2015 |
| Asterionella | Bacillariophyta | 1.74 | Star-shaped colonies, long setae; John et al. 2022 |
| Trachelomonas | Euglenophyta | 1.56 | Loricate cells with anterior collar and apical pore; Hu & Wei 2006 |
| Coelastrum | Chlorophyta | 1.41 | Hollow spherical coenobia with 8–16 cells; Wehr et al. 2015 |
| Ulothrix | Chlorophyta | 1.28 | Unbranched filaments, parietal chloroplast; John et al. 2022 |
| Volvox | Chlorophyta | 1.20 | Hollow spheroids of >500 cells; Hu & Wei 2006 |
| Kirchneriella | Chlorophyta | 1.12 | Small spherical cells in loose mucilage; Wehr et al. 2015 |
| Oocystis | Chlorophyta | 1.05 | Ovoid cells (3–8) in mucilaginous envelope; John et al. 2022 |
| Tetraedron | Chlorophyta | 0.97 | Tetrahedral cells with or without spines; Wehr et al. 2015 |
| Closterium | Chlorophyta | 0.91 | Elongated cells with rounded apices; Hu & Wei 2006 |
| Cosmarium | Chlorophyta | 0.84 | Biradiate cells with median constriction; John et al. 2022 |
| Staurastrum | Chlorophyta | 0.79 | Star-shaped semicells, spines present; Wehr et al. 2015 |
| Phacus | Euglenophyta | 0.72 | Flattened discoid cells, short flagellum; Hu & Wei 2006 |
| Gonium | Chlorophyta | 0.68 | Flat plate of 4–16 cells in square array; John et al. 2022 |
| Crucigenia | Chlorophyta | 0.63 | Cross-shaped coenobia of 4 cells; Wehr et al. 2015 |
| Characium | Chlorophyta | 0.56 | Sessile oval cells attached to substrate; John et al. 2022 |
| Ankistrodesmus | Chlorophyta | 0.52 | Curved needle-like cells in fasciculate groups; Wehr et al. 2015 |
| Nitzschia | Bacillariophyta | 0.48 | Fusiform valves, keel with fibulae; Hu & Wei 2006 |

Note:

Relative abundance = (cell count of the genus / total cell count) × 100 %.

Morphological descriptions follow GB 17378.7-2007, HJ 897-2017, Wehr et al. (2015), and John et al. (2022).

| **Table S3 The phytoplankton cell density of each sample (10^6^ cells/L)** | | | | | | | | | | | | | | | | |
| --- | --- | --- | --- | --- | --- | --- | --- | --- | --- | --- | --- | --- | --- | --- | --- | --- |
|  |  | M | | | | | L | | | | | H | | | | |
|  |  | NQ | HZ | SQ | AK | CXY | CW | NS | LHK | XY | SY | QJ | HC | XT | YC | ZY |
| Phylum | Genus |  |  |  |  |  |  |  |  |  |  |  |  |  |  |  |
| *Cyanophyta* | *Microsystis sp.* | 0.0 | 0.0 | 380.0 | 0.0 | 0.0 | 0.0 | 0.0 | 0.0 | 0.0 | 0.0 | 0.0 | 0.0 | 0.0 | 0.0 | 0.0 |
|  | *Lyngbya limnetica* | 0.0 | 0.0 | 0.0 | 0.0 | 0.0 | 0.0 | 0.0 | 0.0 | 0.0 | 0.0 | 63.3 | 16.7 | 0.0 | 23.3 | 60.0 |
|  | *Dolichospermum sp.* | 0.0 | 0.0 | 0.0 | 0.0 | 0.0 | 0.0 | 0.0 | 0.0 | 0.0 | 0.0 | 0.0 | 0.0 | 0.0 | 0.0 | 0.0 |
|  | *Chroococcus sp.* | 0.0 | 0.0 | 0.0 | 0.0 | 0.0 | 36.7 | 0.0 | 0.0 | 0.0 | 3.3 | 3.3 | 0.0 | 0.0 | 23.3 | 0.0 |
|  | *Anabaena sp.* | 0.0 | 0.0 | 0.0 | 0.0 | 0.0 | 200.0 | 0.0 | 0.0 | 0.0 | 0.0 | 10.0 | 0.0 | 0.0 | 0.0 | 0.0 |
|  | *Crucigenia sp.* | 0.0 | 0.0 | 0.0 | 0.0 | 0.0 | 26.7 | 0.0 | 0.0 | 0.0 | 0.0 | 0.0 | 0.0 | 0.0 | 0.0 | 0.0 |
|  | *Merismopedia sp.* | 0.0 | 0.0 | 0.0 | 0.0 | 0.0 | 0.0 | 0.0 | 400.0 | 0.0 | 0.0 | 0.0 | 110.0 | 106.7 | 0.0 | 0.0 |
| *Bacillariophyta* | *Cymbella sp.* | 3.3 | 10.0 | 0.0 | 3.3 | 0.0 | 3.3 | 0.0 | 0.0 | 0.0 | 0.0 | 0.0 | 0.0 | 0.0 | 0.0 | 0.0 |
|  | *Gomphonema sp.* | 3.3 | 0.0 | 0.0 | 0.0 | 0.0 | 0.0 | 0.0 | 0.0 | 0.0 | 0.0 | 0.0 | 0.0 | 0.0 | 0.0 | 0.0 |
|  | *Nitzschia sp.* | 0.0 | 0.0 | 0.0 | 0.0 | 0.0 | 0.0 | 0.0 | 0.0 | 0.0 | 0.0 | 0.0 | 3.3 | 0.0 | 0.0 | 5.0 |
|  | *Cyclotella sp.* | 6.7 | 35.0 | 6.7 | 6.7 | 3.3 | 16.7 | 10.0 | 13.3 | 6.7 | 20.0 | 30.0 | 10.0 | 36.7 | 3.3 | 35.0 |
|  | *Melosira sp.* | 0.0 | 210.0 | 0.0 | 0.0 | 0.0 | 0.0 | 0.0 | 13.3 | 0.0 | 23.3 | 20.0 | 20.0 | 70.0 | 0.0 | 15.0 |
|  | *Navicula sp.* | 0.0 | 5.0 | 3.3 | 0.0 | 0.0 | 0.0 | 3.3 | 3.3 | 0.0 | 0.0 | 10.0 | 3.3 | 0.0 | 0.0 | 5.0 |
|  | *Fragilaria sp.* | 0.0 | 0.0 | 3.3 | 0.0 | 0.0 | 0.0 | 0.0 | 0.0 | 0.0 | 0.0 | 3.3 | 0.0 | 0.0 | 0.0 | 0.0 |
|  | *Achnanthes coarctata* | 0.0 | 0.0 | 0.0 | 0.0 | 0.0 | 0.0 | 0.0 | 0.0 | 0.0 | 0.0 | 0.0 | 0.0 | 0.0 | 0.0 | 0.0 |
|  | *Synedra sp.* | 0.0 | 0.0 | 0.0 | 3.3 | 0.0 | 0.0 | 0.0 | 0.0 | 0.0 | 3.3 | 0.0 | 0.0 | 3.3 | 0.0 | 0.0 |
|  | *Diatoma sp.* | 0.0 | 0.0 | 0.0 | 0.0 | 0.0 | 0.0 | 0.0 | 0.0 | 0.0 | 0.0 | 0.0 | 0.0 | 0.0 | 0.0 | 0.0 |
|  | *Cocconeis sp.* | 3.3 | 0.0 | 0.0 | 0.0 | 0.0 | 3.3 | 0.0 | 0.0 | 0.0 | 0.0 | 0.0 | 0.0 | 0.0 | 0.0 | 0.0 |
| *Chlorophyta* | *Cladophora aegagropila (Linn.) Trevisan* | 10.0 | 20.0 | 0.0 | 0.0 | 6.7 | 0.0 | 0.0 | 0.0 | 0.0 | 3.3 | 0.0 | 13.3 | 3.3 | 3.3 | 0.0 |
|  | *Palmella mucosa* | 3.3 | 0.0 | 0.0 | 0.0 | 0.0 | 0.0 | 0.0 | 0.0 | 0.0 | 0.0 | 0.0 | 0.0 | 0.0 | 0.0 | 0.0 |
|  | *Selenastrum westii* | 3.3 | 0.0 | 0.0 | 0.0 | 0.0 | 0.0 | 0.0 | 0.0 | 0.0 | 0.0 | 0.0 | 0.0 | 0.0 | 0.0 | 0.0 |
|  | *Actinastrum fluviatile* | 0.0 | 20.0 | 0.0 | 0.0 | 0.0 | 0.0 | 0.0 | 0.0 | 0.0 | 0.0 | 0.0 | 0.0 | 0.0 | 0.0 | 0.0 |
|  | *Cosmarium sp.* | 0.0 | 0.0 | 3.3 | 3.3 | 0.0 | 0.0 | 0.0 | 0.0 | 0.0 | 0.0 | 0.0 | 0.0 | 0.0 | 0.0 | 0.0 |
|  | *Oocystis sp.* | 0.0 | 0.0 | 0.0 | 0.0 | 0.0 | 30.0 | 0.0 | 0.0 | 0.0 | 0.0 | 0.0 | 0.0 | 0.0 | 0.0 | 0.0 |
|  | *Chlamydomonas sp.* | 0.0 | 0.0 | 0.0 | 0.0 | 3.3 | 0.0 | 0.0 | 0.0 | 0.0 | 0.0 | 0.0 | 0.0 | 0.0 | 3.3 | 0.0 |
|  | *Tetraedron pusillum* | 0.0 | 0.0 | 0.0 | 0.0 | 0.0 | 0.0 | 0.0 | 0.0 | 0.0 | 0.0 | 0.0 | 3.3 | 0.0 | 0.0 | 0.0 |
|  | *Scenedesmus* | 0.0 | 0.0 | 0.0 | 0.0 | 0.0 | 13.3 | 0.0 | 0.0 | 0.0 | 0.0 | 0.0 | 0.0 | 0.0 | 0.0 | 0.0 |
|  | *Chaetomorpha spiralis sp.* | 0.0 | 0.0 | 0.0 | 0.0 | 0.0 | 0.0 | 0.0 | 0.0 | 0.0 | 0.0 | 0.0 | 0.0 | 0.0 | 3.3 | 0.0 |
| *Euglenophyta* | *Euglena sp.* | 0.0 | 45.0 | 3.3 | 6.7 | 0.0 | 0.0 | 0.0 | 0.0 | 10.0 | 0.0 | 0.0 | 0.0 | 3.3 | 0.0 | 0.0 |
|  | *Euglena geniculata* | 0.0 | 0.0 | 0.0 | 0.0 | 3.3 | 0.0 | 0.0 | 0.0 | 0.0 | 0.0 | 0.0 | 0.0 | 0.0 | 0.0 | 0.0 |
| *Pyrrophyta* | *Peridiniales sp.* | 0.0 | 0.0 | 0.0 | 0.0 | 0.0 | 0.0 | 0.0 | 0.0 | 0.0 | 0.0 | 0.0 | 0.0 | 0.0 | 0.0 | 0.0 |
|  | *Ceratium sp.* | 0.0 | 0.0 | 0.0 | 0.0 | 0.0 | 0.0 | 0.0 | 3.3 | 0.0 | 0.0 | 0.0 | 0.0 | 0.0 | 0.0 | 0.0 |
| *Cryptophyta* | *Cryptomonas sp.* | 0.0 | 0.0 | 0.0 | 0.0 | 0.0 | 0.0 | 0.0 | 0.0 | 0.0 | 3.3 | 0.0 | 0.0 | 3.3 | 0.0 | 0.0 |

| **Table S4** Shannon diversity index values of phytoplankton and bacteria in all water samples | | | |
| --- | --- | --- | --- |
| Samples | phytoplankton | bacteria | Trophic Group |
| XY | 1.06 | 4.02 | L |
| LHK | 0.00 | 3.90 | L |
| CW | 0.21 | 3.87 | L |
| NS | 0.33 | 3.71 | L |
| SY | 1.21 | 3.71 | L |
| SQ | 0.47 | 3.68 | M |
| AK | 0.44 | 3.88 | M |
| CXY | 0.37 | 3.89 | M |
| HZ | 1.19 | 3.82 | M |
| NQ | 1.16 | 3.86 | M |
| HC | 1.19 | 3.55 | H |
| QJ | 1.48 | 2.29 | H |
| XT | 1.31 | 3.53 | H |
| YC | 1.06 | 3.98 | H |
| ZY | 1.13 | 3.25 | H |

| **Table S5 Physicochemical parameters and trophic status index (TSI) for each sampling site in the Han River** | | | | | |
| --- | --- | --- | --- | --- | --- |
|  | SD(m) | Chl-a(μg/L) | TN(mg/L) | TP(mg/L) | TSI |
| AK | 0.37 | 1.28 | 2.64 | 0.10 | 53.55 |
| CXY | 0.33 | 0.93 | 3.14 | 0.06 | 51.81 |
| HZ | 0.31 | 1.51 | 2.54 | 0.10 | 54.58 |
| NQ | 0.78 | 1.37 | 2.52 | 0.10 | 50.19 |
| SQ | 0.60 | 6.46 | 1.49 | 0.16 | 56.78 |
| NS | 1.32 | 2.57 | 5.73 | 0.04 | 49.82 |
| YC | 0.70 | 17.81 | 3.40 | 0.12 | 61.61 |
| SY | 0.90 | 1.34 | 1.61 | 0.11 | 48.46 |
| ZY | 0.97 | 9.94 | 6.61 | 0.11 | 60.33 |
| QJ | 0.40 | 10.89 | 6.33 | 0.09 | 63.57 |
| XT | 0.37 | 21.26 | 6.83 | 0.12 | 67.71 |
| HC | 0.31 | 7.77 | 7.01 | 0.11 | 64.67 |
| CW | 0.08 | 1.05 | 0.17 | 0.03 | 45.01 |
| LHK | 1.25 | 3.48 | 4.48 | 0.04 | 49.90 |
| XY | 1.10 | 3.14 | 3.61 | 0.04 | 49.95 |

| **Table S6. Raw physicochemical data for all sampling sites in the Han River Basin.** | | | | | | | | | | | | | | |
| --- | --- | --- | --- | --- | --- | --- | --- | --- | --- | --- | --- | --- | --- | --- |
|  | H(m) | WT（℃） | DO | Cond | PH | ORP | FR(m/s) | Chl-a | COD(MN) (mg/L) | TN (mg/L) | NH4+-N (mg/L) | NO3—N (mg/L) | PO43-（mg/L） | TP (mg/L) |
| AK | 0.37 | 31.67 | 7.65 | 400.53 | 7.92 | 313.90 | 0.24 | 1.28 | 9.81 | 2.64 | 0.89 | 1.25 | 0.01 | 0.05 |
| CXY | 0.33 | 27.57 | 6.82 | 244.87 | 8.21 | 238.60 | 0.33 | 0.93 | 12.83 | 3.14 | 1.14 | 1.70 | 0.01 | 0.06 |
| HZ | 0.40 | 32.10 | 9.19 | 318.95 | 7.96 | 312.21 | 0.32 | 1.51 | 14.39 | 2.54 | 0.57 | 1.35 | 0.01 | 0.10 |
| NQ | 0.92 | 28.80 | 5.01 | 277.33 | 7.86 | 234.80 | 1.09 | 1.37 | 3.66 | 2.52 | 0.32 | 0.87 | 0.03 | 0.10 |
| SQ | 0.60 | 30.37 | 4.63 | 284.07 | 7.65 | 89.77 | 0.27 | 6.46 | 10.91 | 1.49 | 0.49 | 0.79 | 0.02 | 0.06 |
| CW | 0.43 | 27.90 | 5.41 | 230.07 | 7.85 | 257.73 | 0.33 | 1.47 | 13.66 | 4.02 | 1.11 | 2.55 | 0.03 | 0.25 |
| LHK | 2.23 | 27.83 | 8.31 | 257.13 | 8.11 | 215.77 | 0.26 | 1.63 | 5.79 | 2.48 | 0.74 | 1.02 | 0.02 | 0.04 |
| XY | 5.17 | 16.07 | 8.82 | 281.40 | 7.53 | 196.67 | 0.29 | 1.24 | 22.99 | 2.43 | 0.81 | 1.41 | 0.01 | 0.03 |
| NS | 6.00 | 22.53 | 9.61 | 288.63 | 7.66 | 251.20 | 0.29 | 2.73 | 14.43 | 3.73 | 0.44 | 1.17 | 0.01 | 0.04 |
| SY | 4.53 | 28.57 | 8.62 | 342.40 | 7.96 | 230.63 | 1.34 | 17.81 | 27.91 | 1.40 | 0.18 | 1.07 | 0.02 | 0.12 |
| YC | 4.63 | 26.90 | 8.53 | 318.57 | 7.85 | 212.47 | 0.29 | 6.34 | 25.68 | 1.61 | 0.15 | 1.63 | 0.02 | 0.11 |
| ZY | 3.20 | 28.60 | 9.70 | 327.07 | 8.12 | 224.10 | 0.44 | 9.94 | 24.94 | 1.61 | 0.46 | 0.98 | 0.01 | 0.10 |
| QJ | 2.67 | 28.83 | 9.20 | 344.13 | 7.91 | 183.73 | 0.50 | 10.89 | 29.86 | 2.33 | 0.31 | 1.56 | 0.01 | 0.09 |
| XT | 1.63 | 29.87 | 9.95 | 383.37 | 7.92 | 169.10 | 0.35 | 21.26 | 95.87 | 2.83 | 0.68 | 1.80 | 0.01 | 0.12 |
| HC | 7.17 | 29.73 | 8.95 | 367.27 | 7.66 | 212.67 | 0.81 | 7.77 | 59.06 | 3.01 | 0.64 | 1.61 | 0.01 | 0.11 |

**Supplementary File S1**

**Complete Bash + R bioinformatics pipeline for 16S rRNA V3–V4 amplicon sequencing Han River**

# 0. environment ---------------------------------------------------

source ~/.bashrc

module load fastp/0.20.0 usearch/11.0.667 R/4.3.1

# 1. raw-data directory --------------------------------------------

RAW_DIR=raw_fastq

OUT_DIR=analysis

mkdir -p $OUT_DIR/{clean,asv,taxa,stats,figures}

# 2. quality filter / merge ----------------------------------------

for f1 in $RAW_DIR/*_R1.fastq.gz; do

f2=${f1/_R1.fastq.gz/_R2.fastq.gz}

base=$(basename ${f1/_R1.fastq.gz/})

fastp -i $f1 -I $f2 \

-o $OUT_DIR/clean/${base}_R1.fq \

-O $OUT_DIR/clean/${base}_R2.fq \

--detect_adapter_for_pe \

--qualified_quality_phred 20 \

--length_required 200

done

# 3. merge + UNOISE3 ASV ------------------------------------------

usearch -fastq_mergepairs $OUT_DIR/clean/*_R1.fq \

-relabel @ \

-fastqout $OUT_DIR/clean/merged.fq

usearch -fastx_uniques $OUT_DIR/clean/merged.fq \

-fastaout $OUT_DIR/asv/uniques.fa \

-sizeout

usearch -unoise3 $OUT_DIR/asv/uniques.fa \

-zotus $OUT_DIR/asv/zotus.fa \

-minsize 4 \

-tabbedout $OUT_DIR/asv/unoise3_stats.txt

# 4. map reads to ASVs & make table --------------------------------

usearch -otutab $OUT_DIR/clean/merged.fq \

-zotus $OUT_DIR/asv/zotus.fa \

-otutabout $OUT_DIR/asv/asv_table.txt \

-threads 8

# 5. taxonomy (RDP/SILVA 138.1) ------------------------------------

usearch -sintax $OUT_DIR/asv/zotus.fa \

-db /db/silva138.1/silva138.1_v4.fasta \

-strand both \

-tabbedout $OUT_DIR/taxa/taxonomy.txt \

-sintax_cutoff 0.8

## R script (analysis.R)

library(phyloseq); library(vegan); library(igraph); library(psych)

# 1. import --------------------------------------------------------------

asv <- read.table("analysis/asv/asv_table.txt", header=T, row.names=1, sep="\t")

tax <- read.table("analysis/taxa/taxonomy.txt", sep="\t", row.names=1, header=F)

colnames(tax) <- c("Taxon", "Confidence")

meta <- read.csv("meta_han_river.csv", row.names=1)

# 2. build phyloseq object -----------------------------------------------

physeq <- phyloseq(otu_table(as(asv, "matrix"), taxa_are_rows=TRUE),

tax_table(as.matrix(tax)),

sample_data(meta))

# 3. rarefy --------------------------------------------------------------

set.seed(123)

physeq.r <- rarefy_even_depth(physeq, rngseed=123, sample.size=27000)

# 4. alpha-diversity ------------------------------------------------------

alpha <- estimate_richness(physeq.r, measures=c("Shannon", "Observed"))

write.csv(alpha, file="analysis/stats/alpha_diversity.csv")

# 5. beta-diversity (Bray-Curtis) -----------------------------------------

bc_dist <- phyloseq::distance(physeq.r, method="bray")

write.csv(as.matrix(bc_dist), file="analysis/stats/bray_curtis_matrix.csv")

# 6. co-occurrence network ----------------------------------------------

relab <- log10(otu_table(physeq.r) + 1)

net <- corr.test(relab, adjust="fdr", method="spearman")

edge <- which(abs(net$r) >= 0.6 & net$p < 0.05, arr.ind=T)

write.csv(data.frame(from=rownames(relab)[edge[,1]],

to=colnames(relab)[edge[,2]],

r=net$r[edge],

p=net$p[edge]),

file="analysis/stats/network_edges.csv")

# 7. RDA/VPA --------------------------------------------------------------

env <- sample_data(physeq.r)[, c("TN","TP","Chl_a","COD","Temp","pH","DO","Cond")]

rda1 <- rda(otu_table(physeq.r) ~ ., data=env, scale=TRUE)

write.csv(summary(rda1)$cont, file="analysis/stats/rda_summary.csv")

# 8. save phyloseq object -------------------------------------------------

saveRDS(physeq.r, "analysis/stats/physeq_final.rds")

raw_fastq/

analysis/

├─ clean/

│ ├─ *_R1.fq / *_R2.fq

│ └─ merged.fq

├─ asv/

│ ├─ zotus.fa

│ ├─ asv_table.txt

│ └─ unoise3_stats.txt

├─ taxa/

│ └─ taxonomy.txt

├─ stats/

│ ├─ alpha_diversity.csv

│ ├─ network_edges.csv

│ ├─ rda_summary.csv

│ └─ physeq_final.rds

└─ figures/
